# Supplementary figures and images for: Cross-regulation and cross-talk of conserved and accessory two-component regulatory systems orchestrate Pseudomonas copper resistance
Source: PLoS Genet. 2024 Jun 11;20(6):e1011325. doi: 10.1371/journal.pgen.1011325 (PMC11195947; doi:10.1371/journal.pgen.1011325)

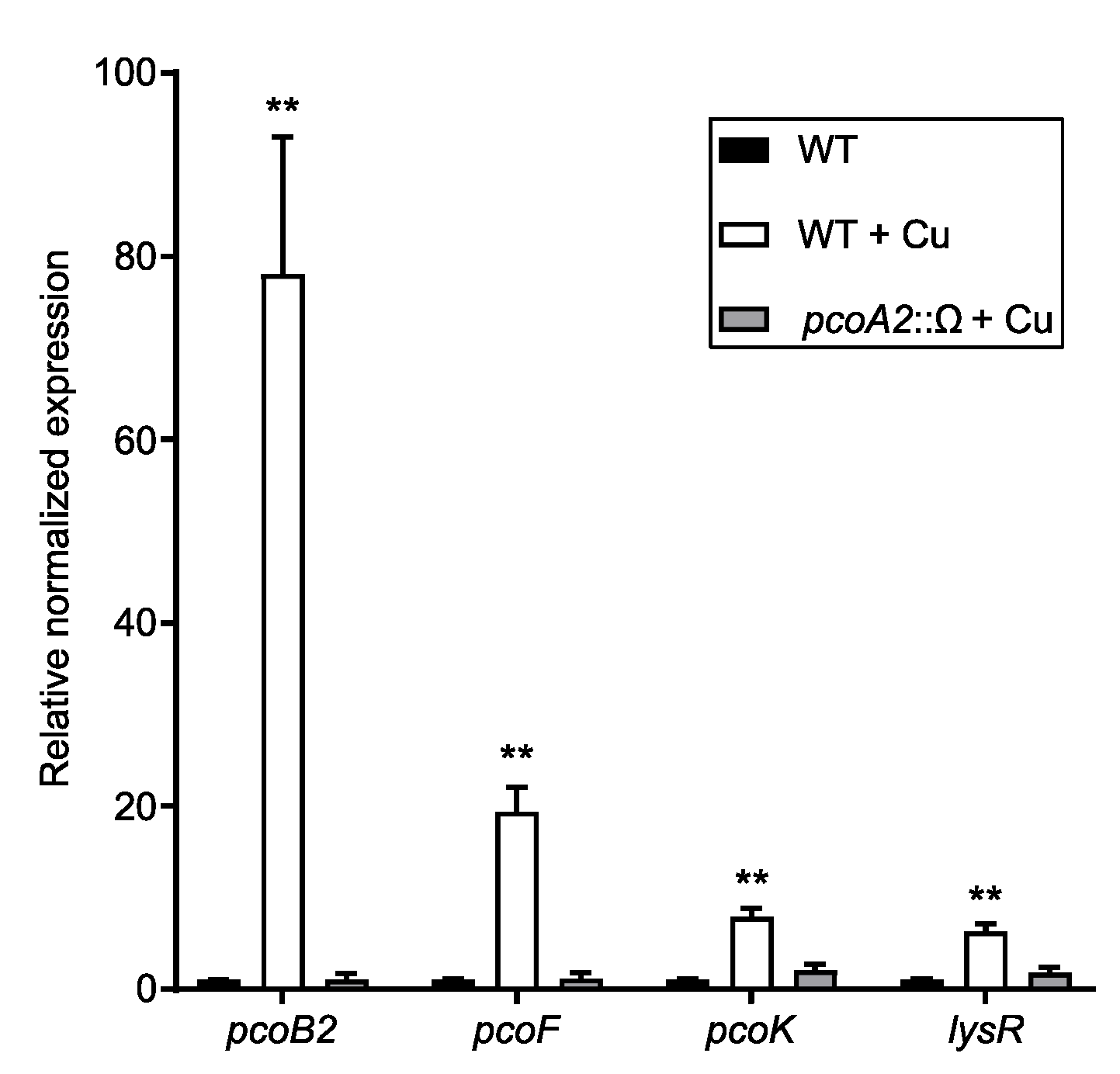

Supplement: S1 Fig — RT-qPCR analysis of relative pcoB2, pcoF, pcoK, and lysR expression in IHMA87 (WT) and IHMA87 pcoA2::Ω. Strains were grown for 2.5 h in LB or LB containing 0.5 mM CuS04, as indicated. The rpoD gene was used as a reference. Experiments were performed in triplicate, and error bars represent the SEM. Significant differences with WT in LB according to unpaired t-test are annotated, ** p-value<0.01. (TIF) [file pgen.1011325.s001.tif]

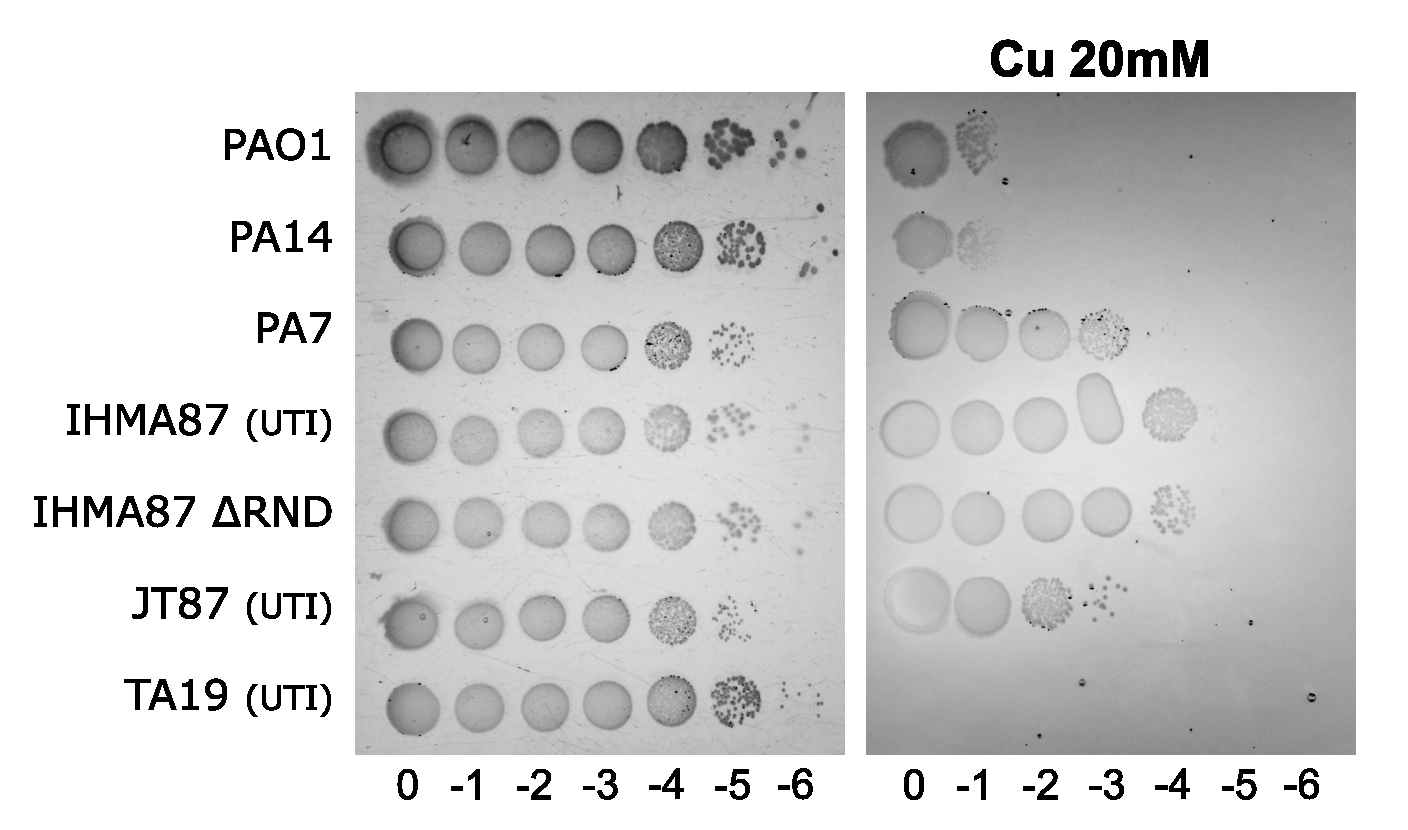

Supplement: S2 Fig — Copper sensitivity plate assays with the strains indicated. 10 μl of 10-fold serial dilutions, as indicated under the images (100 to 10−6), of each strain were deposited on M9 plates and M9 plates supplemented with 20 mM CuSO4. (TIF) [file pgen.1011325.s002.tif]

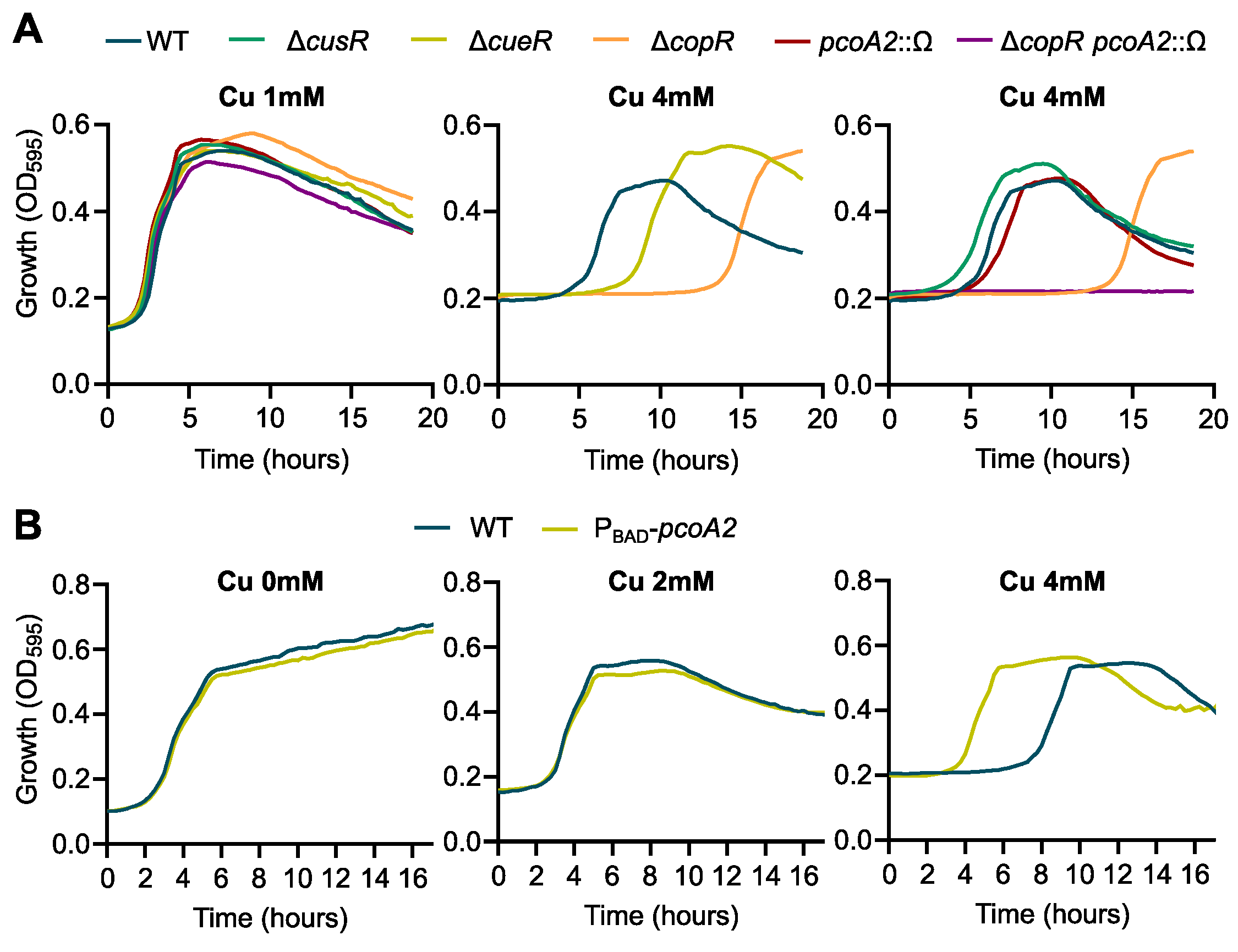

Supplement: S3 Fig — (A) Growth of the different strains was monitored in LB supplemented with various CuSO4 concentrations, as indicated. (B) The PBAD promoter was introduced upstream of the pcoA2 operon to place it under the control of the inducible promoter. The growth of the resulting strain was compared to that of the wild-type strain in LB supplemented with 2% arabinose (to induce PBAD) and 0, 2, or 4 mM CuSO4. (A,B) Growth in 96-well plates was monitored for the times indicated. (TIF) [file pgen.1011325.s003.tif]

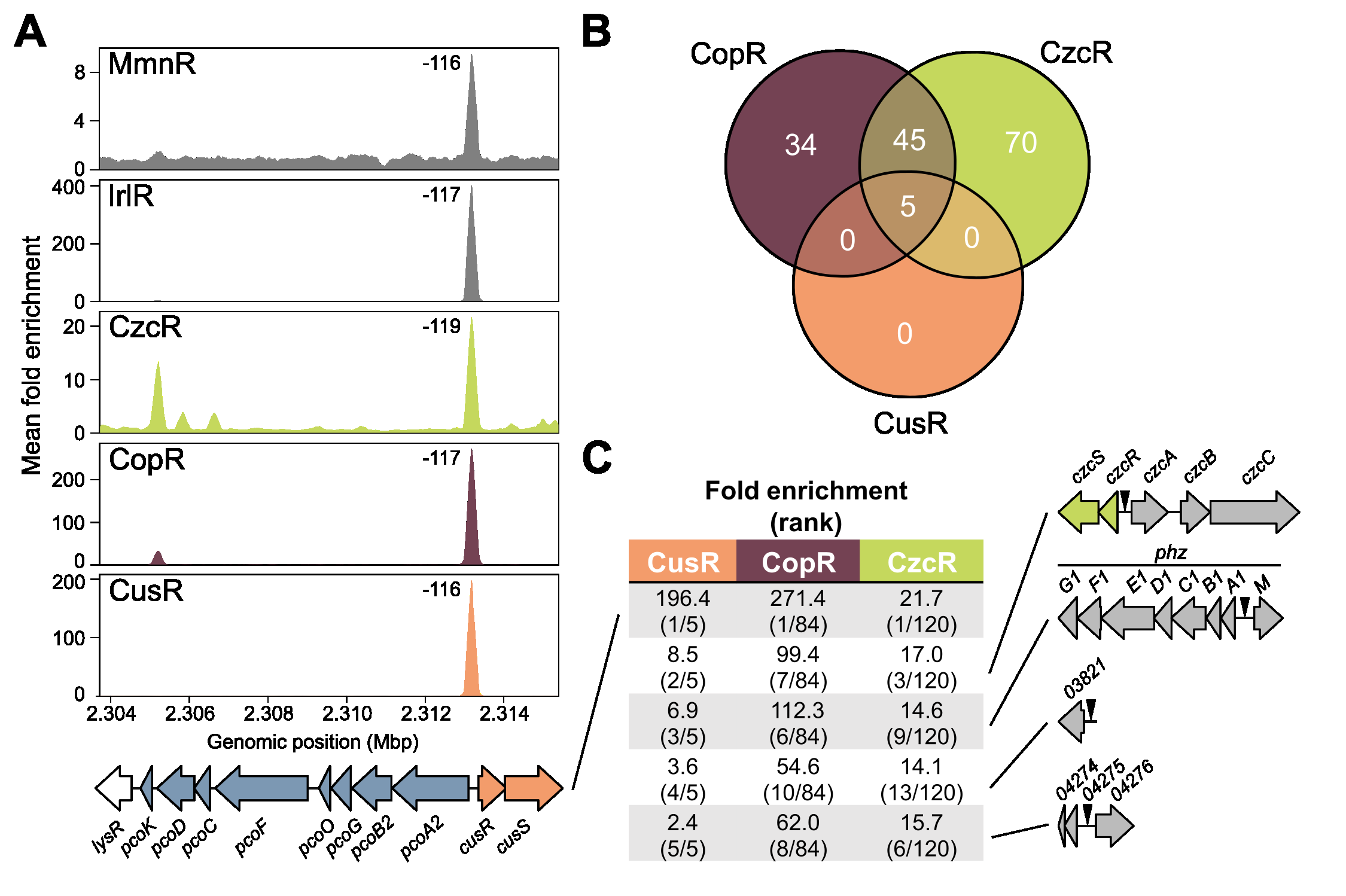

Supplement: S4 Fig — Reanalysis of previously published DAPseq data obtained for the IHMA87 genome [29]. (A) Enrichment coverage tracks of DAP-seq against negative controls are shown for the five RRs with binding sites on the copper-related locus, as indicated. The position of the summit of each peak from the translational start of cusR is given. (B) Venn diagram showing a large range of targets that overlap in vitro for the three RRs on the IHMA87 genome. Each target corresponds to the upstream region of one or more transcriptional units. Boundaries set for the upstream region were -400 and +20 bp from the ATG. (C) Targets common to the 3 RRs on the IHMA87 genome with the fold-enrichment of each peak and its rank among other targets of the RR. The targeted intergenic region of the different transcriptional units is indicated by a black triangle. The annotations “03821” and “04274–76” correspond to “IHMA87_03821” and “IHMA87_04274_76” (www.pseudomonas.com; [28]). (TIF) [file pgen.1011325.s004.tif]

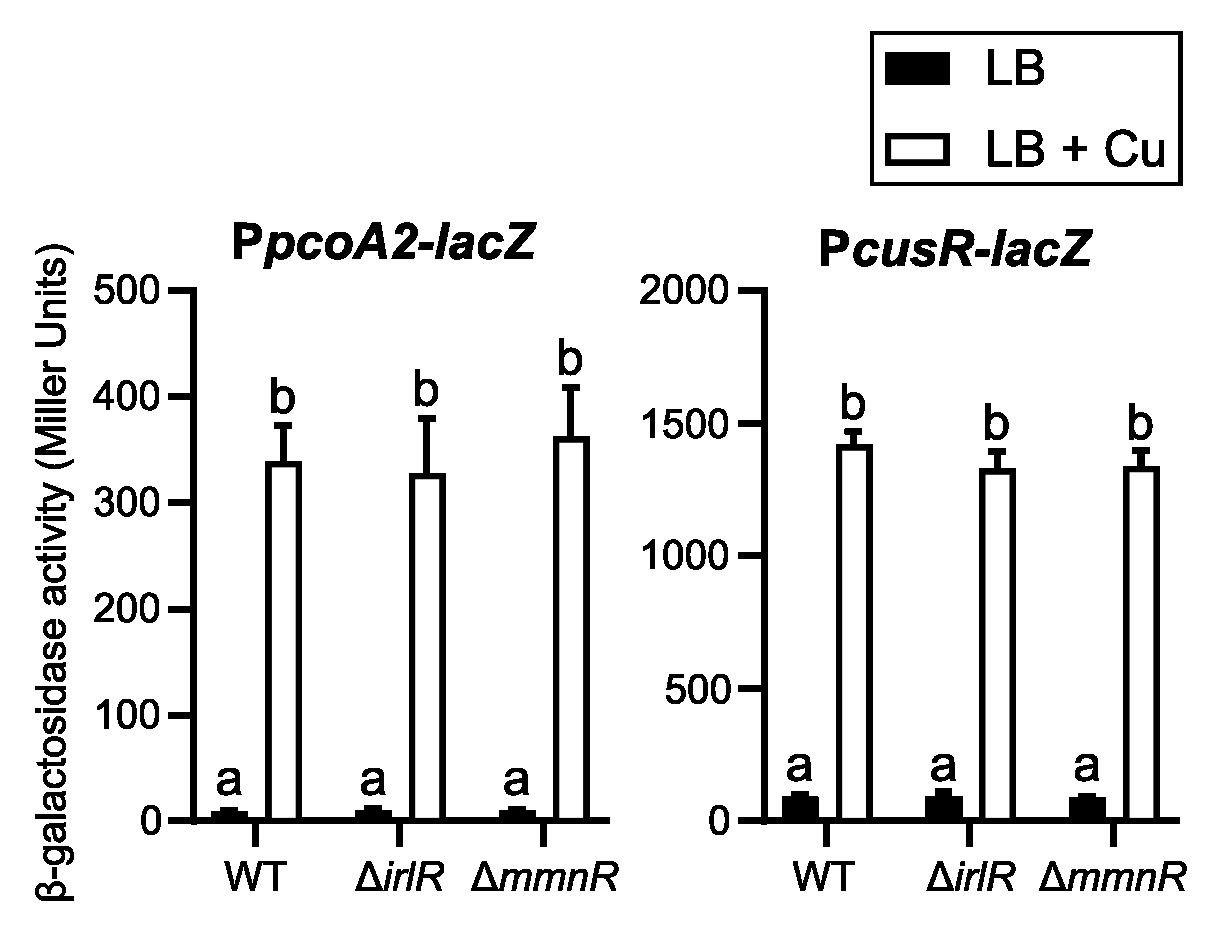

Supplement: S5 Fig — β-galactosidase activities of the indicated strains harboring the PpcoA2-lacZ or PcusR-lacZ transcriptional fusions, after 2.5 h in LB or LB supplemented with 0.5 mM CuSO4. Experiments were performed in triplicate; error bars correspond to SEM. Different letters indicate significant differences according to two-way ANOVA followed by Tukey’s multiple comparison test (p-value < 0.05). (TIF) [file pgen.1011325.s005.tif]

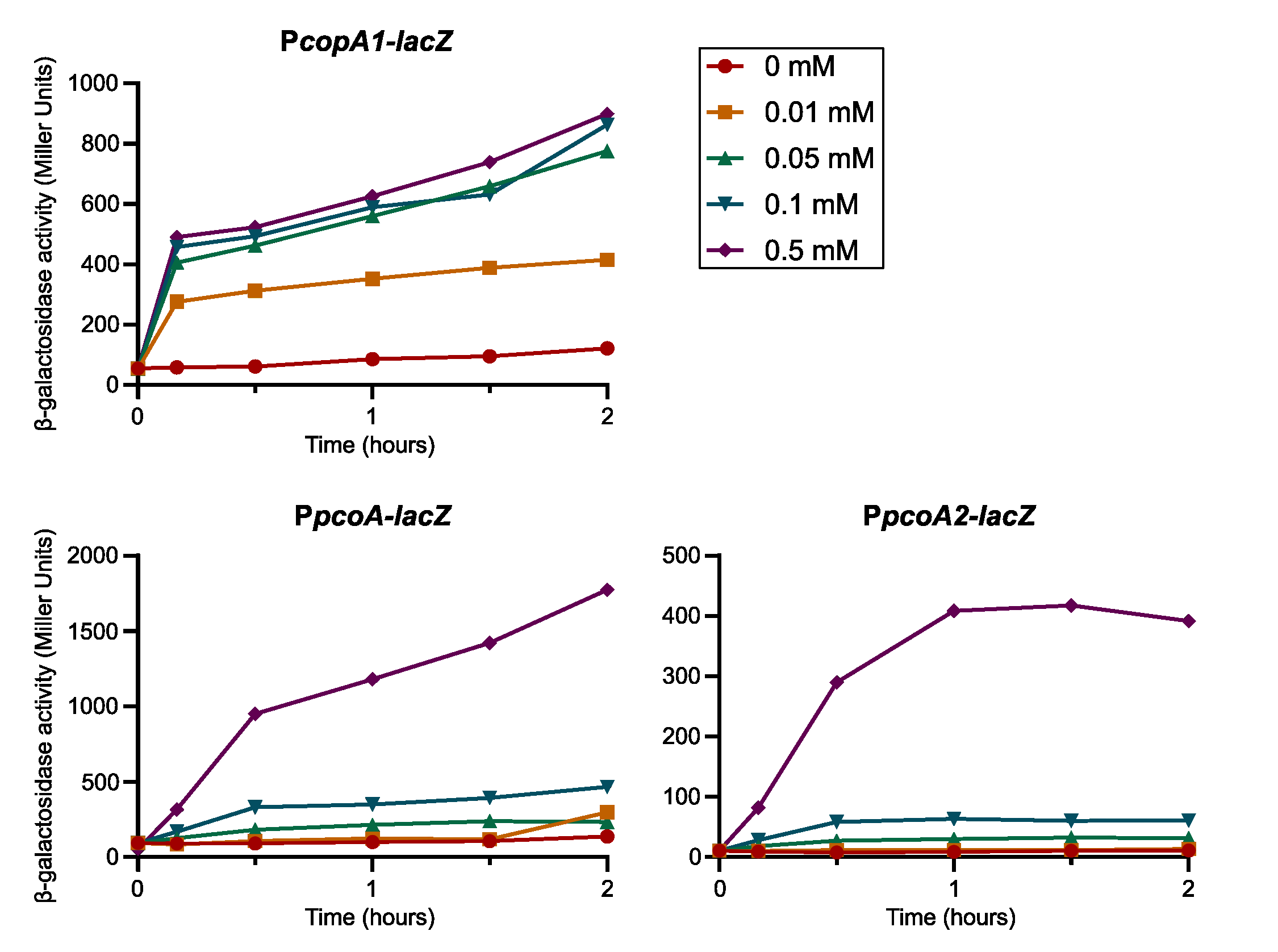

Supplement: S6 Fig — β-galactosidase activities of the wild-type IHMA87 harboring the PcopA1-lacZ, PpcoA-lacZ, or PpcoA2-lacZ transcriptional fusions. Growth in 96-well plates was assessed in LB supplemented with CuSO4 at the concentrations indicated. (TIF) [file pgen.1011325.s006.tif]
