# Supplementary material for: Cross-regulation and cross-talk of conserved and accessory two-component regulatory systems orchestrate Pseudomonas copper resistance
Source: PLoS Genet. 2024 Jun 11;20(6):e1011325. doi: 10.1371/journal.pgen.1011325 (PMC11195947; doi:10.1371/journal.pgen.1011325)
Supplement: S1 Table — (DOCX) [file pgen.1011325.s007.docx]

| **Strain or plasmid** | **Genotype or relevant properties** | **Reference/Source** |
| --- | --- | --- |
| **Strains** |  |  |
| ***P. paraeruginosa*** |  |  |
| IHMA879472 (IHMA87) | wild-type strain (urinary infection) | [1] |
| IHMA87 *copS*_H235A_ | IHMA87 *bearing gene encoding CopS_H235A_ variant* | This study |
| IHMA87 *copS*_T239A_ | IHMA87 *bearing gene encoding CopS_T239A_ variant* | This study |
| IHMA87 *cusS*_T270A_ | IHMA87 *bearing gene encoding CusS_T270A_ variant* | This study |
| IHMA87Δ*cusR* | IHMA87 with *IHMA87_02165* (*cusR*) deletion | This study |
| IHMA87Δ*cusS* | IHMA87 with *IHMA87_02166* (*cusS*) deletion | This study |
| IHMA87Δ*cusRS* | IHMA87 with *IHMA87_02165-02166* deletion | This study |
| IHMA87Δ*copR* | IHMA87 with *IHMA87_02262* (*copR*) deletion | This study |
| IHMA87Δ*copS* | IHMA87 with *IHMA87_02261* (*copS*) deletion | This study |
| IHMA87Δ*copRS* | IHMA87 with *IHMA87_02262-02261 (copRS)* deletion | This study |
| IHMA87Δ*cueR* | IHMA87 with *IHMA87_05258* (*cueR*) deletion | This study |
| IHMA87Δ*cusS*Δ*copS* | IHMA87 with *cusS* and *copS* deletions | This study |
| IHMA87Δ*cusS*Δ*copR* | IHMA87 with *cusS* and *copR* deletions | This study |
| IHMA87Δ*cusS*Δ*copRS* | IHMA87 with *cusS* and *copRS* deletions | This study |
| IHMA87Δ*cusR*Δ*copR* | IHMA87 with *cusR* and *copR* deletions | This study |
| IHMA87Δ*cusR*Δ*cueR* | IHMA87 with *cusR* and *cueR* deletions | This study |
| IHMA87Δ*copR*Δ*cueR* | IHMA87 with *copR* and *cueR* deletions | This study |
| IHMA87Δ*cusR*Δ*copR*Δ*cueR* | IHMA87 with *cusR*, *copR* and *cueR* deletions | This study |
| IHMA87Δ*cusS cusR_D51A_* | IHMA87Δ*cusS bearing gene encoding CusR_D51A_ variant* | This study |
| IHMA87Δ*cusS*Δ*copS cusR_D51A_* | IHMA87Δ*cusS*Δ*copS bearing gene encoding CusR_D51A_ variant* | This study |
| IHMA87Δ*copRS cusR_D51A_* | IHMA87Δ*copRS bearing gene encoding CusR_D51A_ variant* | This study |
| IHMA87Δ*copRS cusS_H266A_* | IHMA87Δ*copRS bearing gene encoding CusS_H266A_ variant* | This study |
| IHMA87Δ*copRS cusS_T270A_* | IHMA87Δ*copRS bearing gene encoding CusS_T270A_ variant* | This study |
| IHMA87Δ*copS*Δ*cusR* | IHMA87 with *copS* and *cusR* deletions | This study |
| IHMA87Δ*copS*Δ*cusRS* | IHMA87 with *copS* and *cusRS* deletions | This study |
| IHMA87Δ*copS cusS_T270A_* | IHMA87Δ*copS bearing gene encoding CusS_T270A_ variant* | This study |
| IHMA87Δ*cusS copS_H235A_* | IHMA87Δ*cusS bearing gene encoding CopS_H235A_ variant* | This study |
| IHMA87Δ*cusS copS_T239A_* | IHMA87Δ*cusS bearing gene encoding CopS_T239A_ variant* | This study |
| IHMA87Δ*cusRS copS_H235A_* | IHMA87Δ*cusRS bearing gene encoding CopS_H235A_ variant* | This study |
| IHMA87Δ*cusRS copS_T239A_* | IHMA87Δ*cusRS bearing gene encoding CopS_T239A_ variant* | This study |
| IHMA87 *::*P*BAD-pcoA2* | IHMA87 with *araC*-P*BAD* inserted upstream *pcoA2* | This study |
| IHMA87 *pcoA2*::Ω | IHMA87 with Ω interposon inserted in *pcoA2* | This study |
| IHMA87Δ*cusR pcoA2*::Ω | IHMA87Δ*cusR* with Ω interposon inserted in *pcoA2* | This study |
| IHMA87Δ*copR pcoA2*::Ω | IHMA87Δ*copR* with Ω interposon inserted in *pcoA2* | This study |
| IHMA87Δ*cusR*Δ*copR pcoA2*::Ω | IHMA87Δ*cusR*Δ*copR* with Ω interposon inserted in *pcoA2* | This study |
| IHMA87Δ*czcR* | IHMA87 with *IHMA87_02627* (*czcR*) deletion | This study |
| IHMA87Δ*irlR* | IHMA87 with *IHMA87_05374* (*irlR*) deletion | This study |
| IHMA87Δ*mmnR* | IHMA87 with *IHMA87_03730* (*mmnR*) deletion | This study |
| IHMA87Δ*RND* | IHMA87 with *IHMA87_02154-02152* deletion | This study |
| ***E. coli*** |  |  |
| TOP10 | Chemically competent cell | Invitrogen |
| BL21 Star (DE3) | F^-^ *ompT hsdS_B_* (r_B_^-^ m_B_^-^) *gal dcm rne131*(DE3) | Invitrogen |
|  |  |  |
| **Plasmids** |  |  |
| pRK600 | Helper plasmid with conjugative properties (Cm^R^ ) | [2] |
| pEXG2 | Allelic exchange vector (Gm^R^ ) | [3] |
| pEXG2-IHMAΔ*cusR* | pEXG2 carrying SLIC fragment for *cusR* deletion (Gm^R^ ) | This study |
| pEXG2-IHMA *cusR D51A* | pEXG2 carrying SLIC fragment for *cusR* mutation (Gm^R^ ) | This study |
| pEXG2-IHMAΔ*cusS bis* | pEXG2 carrying SLIC fragment for *cusS* deletion (Gm^R^ ) | This study |
| pEXG2-IHMA*cusS*_H266A | pEXG2 carrying SLIC fragment for *cusS* mutation (Gm^R^ ) | This study |
| pEXG2-IHMA*cusS*_T270A | pEXG2 carrying SLIC fragment for *cusS* mutation (Gm^R^ ) | This study |
| pEXG2-IHMAΔ*cusRS* | pEXG2 carrying SLIC fragment for *cusRS* deletion (Gm^R^ ) | This study |
| pEXG2-IHMAΔ*copR* | pEXG2 carrying SLIC fragment for *copR* deletion (Gm^R^ ) | This study |
| pEXG2-IHMAΔ*copS bis* | pEXG2 carrying SLIC fragment for *copS* deletion (Gm^R^ ) | This study |
| pEXG2-IHMA*copS*_H235A | pEXG2 carrying SLIC fragment for *copS* mutation (Gm^R^ ) | This study |
| pEXG2-IHMA*copS*_T239A | pEXG2 carrying SLIC fragment for *cusS* mutation (Gm^R^ ) | This study |
| pEXG2-IHMAΔ*copRS bis* | pEXG2 carrying SLIC fragment for *copRS* deletion (Gm^R^ ) | This study |
| pEXG2-IHMAΔ*cueR* | pEXG2 carrying SLIC fragment for *cueR* deletion (Gm^R^ ) | This study |
| pEXG2-IHMAΔ*czcR* | pEXG2 carrying SLIC fragment for *czcR* deletion (Gm^R^ ) | This study |
| pEXG2-IHMAΔ*irlR* | pEXG2 carrying SLIC fragment for *irlR* deletion (Gm^R^ ) | This study |
| pEXG2-IHMAΔ*mmnR* | pEXG2 carrying SLIC fragment for *mmnR* deletion (Gm^R^ ) | This study |
| pEXG2-IHMAΔ*RND* | pEXG2 carrying SLIC fragment for *IHMA87_02154-02153-02152* deletion (Gm^R^ ) | This study |
| pEXG2-P*pcoA2-*Sp | pEXG2 carrying SLIC fragment for *araC-*P*BAD* insertion upstream of the  *pcoA2* operon (Gm^R^) | This study |
| pEXG2-P*BAD*-*pcoA2*-Sp | pEXG2-P*pcoA2-*Sp with *araC*-P*BAD* fragment from pSW196 (Gm^R^) | This study |
| pSW196 | Site-specific integrative plasmid with P*BAD30*-promoter (*attP* site, FRT, *Tc^R^*) | [4] |
| pEXG2ΔB-*pcoA2* | *Bam*HI-deleted pEXG2 carrying 995-bp fragment of *pcoA2* gene (Gm^R^) | This study |
| pEXG2ΔB-Ω-*pcoA2* | pEXG2ΔB-*pcoA2* with Ω interposon inserted into *pcoA2* fragment (Gm^R^, Spc^R^, Sm^R^) | This study |
| pHP45Ω | Derivative of pBR322 plasmid carrying the Ω interposon (Ap^R^, Spc^R^, Sm^R^) | [5] |
| miniCTX1-lacZ | Site-specific integrative plasmid with promoter-less *lacZ* (*attP* site, FRT, *Tc^R^*) | [6] |
| miniCTX-T*rrnB*-*lacZ* | mini-CTX1-lacZ with strong *rrnB* terminator (*attP*, FRT, *Tc^R^*) | This study |
| pCTXter-P*cusR*-*lacZ* | miniCTX-TrrnB-lacZ harboring the *cusR* promoter (503 bp) fused to *lacZ* (*attP*,Tc^R^) | This study |
| pCTXter-P*cusR2*-*lacZ* | miniCTX-TrrnB-lacZ harboring the *cusR* promoter (126 bp) fused to *lacZ* (*attP*,Tc^R^) | This study |
| pCTXter-P*cusR3*-*lacZ* | miniCTX-TrrnB-lacZ harboring the *cusR* promoter (85 bp) fused to *lacZ* (*attP*,Tc^R^) | This study |
| pCTXter-P*cusR4*-*lacZ* | miniCTX-TrrnB-lacZ harboring the *cusR* promoter (144 bp) fused to *lacZ* (*attP*,Tc^R^) | This study |
| pCTXter-P*cusR*-*DBSmut-lacZ* | miniCTX-TrrnB-lacZ harboring the mutated *cusR* promoter (503 bp) fused to *lacZ* (*attP*,Tc^R^) | This study |
| pCTXter-*PpcoA2*-lacZ | miniCTX-TrrnB-lacZ harboring the *pcoA2* promoter (501 bp) fused to *lacZ* (*attP*,Tc^R^) | This study |
| pCTXter-P*pcoA2 sh-lacZ* | miniCTX-TrrnB-lacZ harboring the *pcoA2* promoter (171 bp) fused to *lacZ* (*attP*,Tc^R^) | This study |
| pCTXter-*PpcoA2 DBSmut*-*lacZ* | miniCTX-TrrnB-lacZ harboring the mutated *pcoA2*  promoter (501 bp) fused to *lacZ* (*attP*,Tc^R^) | This study |
| pCTXter-*PcopR*-*lacZ* | miniCTX-TrrnB-lacZ harboring the *copR* promoter fused to *lacZ* (*attP*,Tc^R^) | This study |
| pCTXter-P*copA1*-*lacZ* | miniCTX-TrrnB-lacZ harboring the *copA1* promoter fused to *lacZ* (*attP*,Tc^R^) | This study |
| pCTXter-IHMA-P*pcoA-lacZ* | miniCTX-TrrnB-lacZ harboring the *pcoA* promoter fused to *lacZ* (*attP*,Tc^R^) | This study |
| pCTXter-P*rhlA-lacZ* | miniCTX-TrrnB-lacZ harboring the *rhlA* promoter fused to *lacZ* (*attP*,Tc^R^) | This study |
| miniCTX-P*czcCBA-lacZ* | miniCTX-lacZ harboring the *czcA* promoter fused to *lacZ* (*attP*,Tc^R^) | This study |
| pET15b | Expression vector (Ap^R^ ) | Novagen |
| pET15b-CusR | Expression vector of His6-CusR (Ap^R^ ) | This study |

**Supplementary references**

1. Kos VN, Déraspe M, McLaughlin RE, Whiteaker JD, Roy PH, Alm RA, et al. The Resistome of *Pseudomonas aeruginosa* in Relationship to Phenotypic Susceptibility. Antimicrob Agents Chemother. 2015;59: 427–436. doi:10.1128/AAC.03954-14

2. Kessler B, de Lorenzo V, Timmis KN. A general system to integrate *lacZ* fusions into the chromosomes of gram-negative eubacteria: regulation of the Pm promoter of theTOL plasmid studied with all controlling elements in monocopy. Molec Gen Genet. 1992;233: 293–301. doi:10.1007/BF00587591

3. Rietsch A, Vallet-Gely I, Dove SL, Mekalanos JJ. ExsE, a secreted regulator of type III secretion genes in *Pseudomonas aeruginosa*. Proc Natl Acad Sci USA. 2005;102: 8006–8011. doi:10.1073/pnas.0503005102

4. Baynham PJ, Ramsey DM, Gvozdyev BV, Cordonnier EM, Wozniak DJ. The *Pseudomonas aeruginosa* Ribbon-Helix-Helix DNA-Binding Protein AlgZ (AmrZ) Controls Twitching Motility and Biogenesis of Type IV Pili. J Bacteriol. 2006;188: 132–140. doi:10.1128/JB.188.1.132-140.2006

5. Prentki P, Krisch HM. In vitro insertional mutagenesis with a selectable DNA fragment. Gene. 1984;29: 303–313. doi:10.1016/0378-1119(84)90059-3

6. Becher A, Schweizer HP. Integration-Proficient *Pseudomonas aeruginosa* Vectors for Isolation of Single-Copy Chromosomal *lacZ* and *lux* Gene Fusions. BioTechniques. 2000;29: 948–952. doi:10.2144/00295bm04
