# Supplementary material for: Cross-regulation and cross-talk of conserved and accessory two-component regulatory systems orchestrate Pseudomonas copper resistance
Source: PLoS Genet. 2024 Jun 11;20(6):e1011325. doi: 10.1371/journal.pgen.1011325 (PMC11195947; doi:10.1371/journal.pgen.1011325)
Supplement: S2 Table — (DOCX) [file pgen.1011325.s008.docx]

| **Name** | **Sequence (5’ -> 3’)** | **Use** |
| --- | --- | --- |
| **pEXG2-mut-cusR-sF1** | GGTCGACTCTAGAGGATCCCC GTAGCCACGCCTGGCTTG | *cusR* deletion |
| **pEXG2-mut-cusR-sR1-bis** | GAGCGTGTATCCCATTCCACG CAGCAGTTTCATCGCATGTCC | *cusR* deletion |
| **pEXG2-mut-cusR-sF2** | GTGGAATGGGATACACGCTCG | *cusR* deletion |
| **pEXG2-mut-cusR-sR2** | ACCGAATTCGAGCTCGAGCCC CGCTGAATCGTCAGCAGGCA | *cusR* deletion |
| **pEXG2-mut-cusR-sF1** | GGTCGACTCTAGAGGATCCCC GTAGCCACGCCTGGCTTG | CusR_D51A_ mutation |
| **pEXG2-cusR-D51A-sR1** | GG CCAAGACCACCAGATCGTATGT | CusR_D51A_ mutation |
| **pEXG2-cusR-D51A-sF2** | TACGATCTGGTGGTCTTGGCC GTCATGCTGCCGGATGTCAATG | CusR_D51A_ mutation |
| **pEXG2-mut-cusR-sR2** | ACCGAATTCGAGCTCGAGCCC CGCTGAATCGTCAGCAGGCA | CusR_D51A_ mutation |
| **pEXG2-mut-cusS-sF1** | GGTCGACTCTAGAGGATCCCC GCGCTGGCCAACGCATCAAT | *cusS* deletion |
| **pEXG2-mut-cusS-sR1** | TCA GGCCAAGTGTTGCTTCAGTTC | *cusS* deletion |
| **pEXG2-mut-cusS-sF3** | CTGAAGCAACACTTGGCCTGA ATTGCGGAGGCACATGGTGGC | *cusS* deletion |
| **pEXG2-mut-cusS-sR3** | ACCGAATTCGAGCTCGAGCCC AGTACCTTGATCTTGATCAATAAG | *cusS* deletion |
| **pEXG2-cusS-H266A-sF1** | GGTCGACTCTAGAGGATCCCC ATTTGATCGTGTGGCAGGAGGAT | CusS_H266A_ mutation |
| **pEXG2-cusS-H266A-sR1** | CGCGGCGATGTCTGCGGAAAAGTGTG | CusS_H266A_ mutation |
| **pEXG2-cusS-H266A-sF2** | TTTTCCGCAGACATCGCCGCG GAGTTGCGCACGCCCGTCAC | CusS_H266A_ mutation |
| **pEXG2-cusS-H266A-sR2** | ACCGAATTCGAGCTCGAGCCC GTCCTGCGAAAGCAAGACCG | CusS_H266A_ mutation |
| **pEXG2-cusS-H266A-sF1** | GGTCGACTCTAGAGGATCCCC ATTTGATCGTGTGGCAGGAGGAT | CusS_T270A_ mutation |
| **pEXG2-cusS-T270A-sR1** | CGCGCAACTCGTGCGCGATGT | CusS_T270A_ mutation |
| **pEXG2-cusS-T270A-sF2** | CATCGCGCACGAGTTGCGCG CGCCCGTCACCAACCTGGTC | CusS_T270A_ mutation |
| **pEXG2-cusS-T270A-sR2** | ACCGAATTCGAGCTCGAGCCC ACTGATCGTTGTCCACTGCTC | CusS_T270A_ mutation |
| **pEXG2-mut-cusRS-sF1** | GGTCGACTCTAGAGGATCCCC GTTGGTTACACGGATTGTGACG | *cusRS* deletion |
| **pEXG2-mut-cusRS-sR1** | TCA GTTCTCGTCTTCAACGACCAGC | *cusRS* deletion |
| **pEXG2-mut-cusRS-sF2** | GTCGTTGAAGACGAGAACTGA CAGGACGAGCAGTGGACAACG | *cusRS* deletion |
| **pEXG2-mut-cusRS-sR2** | ACCGAATTCGAGCTCGAGCCC GCGCAACGAACCAGCAG | *cusRS* deletion |
| **pEXG2-mut-IHMA copR-sF1** | GGTCGACTCTAGAGGATCCCC AAACCCGCCGACCGGAAACCA | *copR* deletion |
| **pEXG2-mut-IHMA copR-sR1** | TCATTCGTCGCGTTCTTCGAG GATCAGCAGTTTCATGTTCATCC | *copR* deletion |
| **pEXG2-mut-IHMA copR-sF2** | CTCGAAGAACGCGACGAATGAG | *copR* deletion |
| **pEXG2-mut-IHMA copR-sR2** | ACCGAATTCGAGCTCGAGCCC TGATGTCGAGGATCACGGTGAG | *copR* deletion |
| **pEXG2-mut-copS-sF1** | GGTCGACTCTAGAGGATCCCC GGTGCTGTTCCTCACTGC | *copS* deletion |
| **pEXG2-mut-copS-sR1** | TCA GGATAGCCGGGAACCGAGTCC | *copS* deletion |
| **pEXG2-mut-copS-sF2** | CTCGGTTCCCGGCTATCCTGA GAGATCCGCTGCGAGTCCGC | *copS* deletion |
| **pEXG2-mut-copS-sR2** | ACCGAATTCGAGCTCGAGCCC AAACTGCAGGTACACCACCTG | *copS* deletion |
| **pEXG2-copS-H235A-sF1** | GGTCGACTCTAGAGGATCCCC GCCTGCCGCAGCCTGCGCA | CopS_H235A_ mutation |
| **pEXG2-copS-H235A-sR1** | GCG GGCGATGTCGGCGGAGAACG | CopS_H235A_ mutation |
| **pEXG2-copS-H235A-sF2** | TTCTCCGCCGACATCGCCGCG GAACTGCGCACGCCACTCAC | CopS_H235A_ mutation |
| **pEXG2-copS-H235A-sR2** | ACCGAATTCGAGCTCGAGCCC GGCGGTCGCCGAGGCGGAT | CopS_H235A_ mutation |
| **pEXG2-copS2-T239A-sF1** | GGTCGACTCTAGAGGATCCC C TGGTTCGAGCGCCTGCCGCA | CopS_T239A_ mutation |
| **pEXG2-copS3-T239A-sR1** | AGCAGGCTGGTGAGTGGTGC GCGCAGTTCGTGGGCGATGT | CopS_T239A_ mutation |
| **pEXG2-copS3-T239A-sF2** | GCA CCACTCACCAGCCTGCTGAC | CopS_T239A_ mutation |
| **pEXG2-copS2-T239A-sR2** | ACCGAATTCGAGCTCGAGCCC GAATCGCCGGGCCCTGGTTC | CopS_T239A_ mutation |
| **pEXG2-mut- copR-bis-sF1** | GGTCGACTCTAGAGGATCCCC ATTACATCTTTGTCAGCTTGCC | *copRS* deletion |
| **pEXG2-mut- IHMA copRS-sR1** | TCA GATCAGCAGTTTCATGTTCATCC | *copRS* deletion |
| **pEXG2-mut- IHMA copRS-sF4** | AACATGAAACTGCTGATCTGA CGCCTGAGCGTGGAGAACCA | *copRS* deletion |
| **pEXG2-mut-copRS-sR4** | ACCGAATTCGAGCTCGAGCCC TGCTGACGCCGCTGACCTAC | *copRS* deletion |
| **pEXG2-mut-IHMA cueR-sF1** | GGTCGACTCTAGAGGATCCCC GATGGTCGGCAACCTGGTGG | *cueR* deletion |
| **pEXG2-mut-IHMA cueR-sR1** | CCTGACTCCAGGTCCCTGAG GATGTTCATCGGAATCTCCTGG | *cueR* deletion |
| **pEXG2-mut-IHMA cueR-sF2** | CTCAGGGACCTGGAGTCAGG | *cueR* deletion |
| **pEXG2-mut-IHMA cueR-sR2** | ACCGAATTCGAGCTCGAGCCC GGTGTGGGACCGCCTGCAC | *cueR* deletion |
| **pEXG2-mut-IHMA czcR-sF1** | GGTCGACTCTAGAGGATCCCC GATATTCGAAGTTGCACTTCCGG | *czcR* deletion |
| **pEXG2-mut-IHMA czcR-sR1** | GACTTCATCTTCGATAATAAGGATG | *czcR* deletion |
| **pEXG2-mut-IHMA czcR-sF2** | CTTATTATCGAAGATGAAGTC GTCGGCTACGTGCTGGAAGC | *czcR* deletion |
| **pEXG2-mut-IHMA czcR-sR2** | ACCGAATTCGAGCTCGAGCCC GATGCTCAGCAGGCGCTGGT | *czcR* deletion |
| **pEXG2-mut-IHMA irlR-sF1** | GGTCGACTCTAGAGGATCCCC TCGAGGGCCTTGAGGGTG | *irlR* deletion |
| **pEXG2-mut-IHMA irlR-sR1** | CGCGTCGTCTTCGACCACCA | *irlR* deletion |
| **pEXG2-mut-IHMA irlR-sF2** | TGGTGGTCGAAGACGACGCG GGCTACGTCTGCGAGGAGCG | *irlR* deletion |
| **pEXG2-mut-IHMA irlR-sR2** | ACCGAATTCGAGCTCGAGCCC GGTGATCTGCAACGGCTCG | *irlR* deletion |
| **pEXG2-mut-IHMA mmnR-sF1** | GGTCGACTCTAGAGGATCCCC AGCGCGAGCAGGGCCTGAG | *mmnR* deletion |
| **pEXG2-mut-IHMA mmnR-sR1** | CGCCTCGTCCTCGACTATCAG | *mmnR* deletion |
| **pEXG2-mut-IHMA mmnR-sF2** | TGATAGTCGAGGACGAGGCG CATACCATCCGGGGCATGGG | *mmnR* deletion |
| **pEXG2-mut-IHMA mmnR-sR2** | ACCGAATTCGAGCTCGAGCCC GGTGTTCCGGCAACTGCAACT | *mmnR* deletion |
| **pEXG2-mut-IHMA RND-sF1** | GGTCGACTCTAGAGGATCCCC gtacggccgaggcacaacg | RND genes deletion |
| **pEXG2-mut-IHMA RND-sR1** | GATCAGCCTGGATCGCCTTGCAGTG | RND genes deletion |
| **pEXG2-mut-IHMA RND-sF2** | gcaaggcgatccaggcTGAtc ggtggccaatcgcttcctgGT | RND genes deletion |
| **pEXG2-mut-IHMA RND-sR2** | ACCGAATTCGAGCTCGAGCCC AAGACCCTGGACGAGTTCC | RND genes deletion |
| **pEXG2-PpcoA2-Sp-sF1** | GTCGACTCTAGAGGATCCCC AAGTCCAGCACCAAATCGGCAA | P*BAD* insertion |
| **pEXG2-PpcoA2-Sp-sR1** | ACTAGT CATCCTGAGCGGCACAGTTCG | P*BAD* insertion |
| **pEXG2-PpcoA2-Sp-sF2** | TGCCGCTCAGGATGACTAGT GGGTGGCGTTTAGAACGACTTC | P*BAD* insertion |
| **pEXG2-PpcoA2-Sp-sR2** | CCGAATTCGAGCTCGAGCCC GCAGCACGTGGTCACGGTCG | P*BAD* insertion |
| **pEXG2_B-omega2-02164-sF1** | GACTCTAGAGGATCGATCCCC GAACATTACATGAATGTAATTCAGG | Insertion interposon Ω in *pcoA2* |
| **pEXG2-omega2-02164-sF1** | TA CCAGTAGGAGCCGCTCTGCT | Insertion interposon Ω in *pcoA2* |
| **pEXG2-omega2-02164-sF2** | AGCAGAGCGGCTCCTACTGGAT CCACTCGCACTCCGGCTTCC | Insertion interposon Ω in *pcoA2* |
| **pEXG2-omega2-02164-sR2** | ACCGAATTCGAGCTCGAGCCC GCGTAGCCTGTCCGGTCCA | Insertion interposon Ω in *pcoA2* |
| **pCTXter-IHMA-cusR-lacZ-sF** | GATATCGAATTCCTGCAGCCC GTTGATGGTCGTAGCCACG | P*cusR-lacZ* fusion |
| **pCTXter-IHMA-cusR-lacZ-sR** | GCTAGTTAGTTAGGATCCCCC ATCGCATGTCCCATGAAATCG | P*cusR-lacZ* fusion |
| **pCTXter-IHMA-cusR2-lacZ-sF** | GATATCGAATTCCTGCAGCCC CATGTAATGTTCGTGTCATGTTC | P*cusR-lacZ* fusion |
| **pCTXter-IHMA-PcusR3-lacZ** | GATATCGAATTCCTGCAGCCC TAAATTGCTGATTGAAAGTGGATGA | P*cusR-lacZ* fusion |
| **pCTXter-IHMAcusR4-lacZ-sF** | GATATCGAATTCCTGCAGCCC TGACCCCTGAATTACATTCATGTA | P*cusR-lacZ* fusion |
| **pCTXter-cusR-BS1-lacZ-sR1** | GAACATGACACGAACAGGCT ATGAATGTAATTCAGGGGTCATTC | P*cusR/pcoA2 DBSmut-lacZ* fusion |
| **pCTXter-cusR-BS1-lacZ-sF2** | AGCCTGTTCGTGTCATGTTCGTGGAA | P*cusR/pcoA2 DBSmut-lacZ* fusion |
| **pCTXter-IHMA_02164-Z-sF** | GATATCGAATTCCTGCAGCCC ACGCGCCGTCAGAAAGAGC | P*pcoA2-lacZ* fusion |
| **pCTXter-IHMA_02164-Z-sR** | GCTAGTTAGTTAGGATCCCCC ATGGCCTGAAATCCTCCATAG | P*pcoA2-lacZ* fusion |
| **pCTXter-IHMA_02164short-Z-sF** | GATATCGAATTCCTGCAGCCC TTGCCAGCTTCCACGAACATGA | P*pcoA2-lacZ* fusion |
| **pCTXter-IHMA-copR-lacZ-sF** | GATATCGAATTCCTGCAGCCC TCGGGAGCATTACATCTTTGTC | *IHMA87_02262-lacZ* fusion |
| **pCTXter-IHMA-copR-lacZ-sR** | GCTAGTTAGTTAGGATCCCCC ATGTTCATCCCTCGTTACATTT | *IHMA87_02262-lacZ* fusion |
| **pCTXter-IHMA-copA1-lacZ-sF** | GATATCGAATTCCTGCAGCCC ATACGCGATCACCTTCTGC | *IHMA87_01025-lacZ* fusion |
| **pCTXter-IHMA-copA1-lacZ-sR** | GCTAGTTAGTTAGGATCCCCC ATGGAACCTCCTTGGAATCG | *IHMA87_01025-lacZ* fusion |
| **pCTXter-IHMA-pcoA-lacZ-sF** | GATATCGAATTCCTGCAGCCC GACCAACTTCGCCGAGAACAT | *IHMA87_0306-lacZ* fusion |
| **pCTXter-IHMA-pcoA-lacZ-sR** | GCTAGTTAGTTAGGATCCCCC ATCCGCTGGTTTCCACGCA | *IHMA87_0306-lacZ* fusion |
| **pCTX-czcCBA-lgl-sF** | GCCCCCCCTCGAGGTCGA ttcgcccctatataaagtatgga | P*czcC-lacZ* fusion |
| **pCTX-czcCBA-lgl-sR** | ATCAAGCTTATCGATACCGTCGA ttccgctcctcgtctgctga | P*czcC-lacZ* fusion |
| **pCTXter-rhlA-lacZ-sF** | GATATCGAATTCCTGCAGCCC GAAGGCCATGTGACCCTCGAG | P*rhlA-lacZ* fusion |
| **pCTXter-rhlA-lacZ-sR** | GCTAGTTAGTTAGGATCCCCC CCGCATTTCACACCTCCCAAAAA | P*rhlA-lacZ* fusion |
| **pET15b-cusR-sF** | ctggtgccgcgcggcagccat ATGAAACTGCTGGTCGTTGAAGA | HMA87 *cusR* overexpression |
| **pET15b-cusR-sR** | GCTTTGTTAGCAGCCGGATCC TTATGGCGCAGGGGCATCATC | HMA87 *cusR* overexpression |
| **rpoDqPCR F1** | GCGCAACAGCAATCTCGTCT | RT-qPCR |
| **rpoDqPCR R1** | ATCCGGGGCTGTCTCGAATA | RT-qPCR |
| **02163_qPCR_up** | AGCCGTCAATGGTTGGCTTT | RT-qPCR |
| **02163_qPCR_down** | CGTCCACCACTTCCCACGTA | RT-qPCR |
| **02160_qPCR_up** | TCGCAGATCGTCCAGATGGT | RT-qPCR |
| **02160_qPCR_down** | CGACGGCCATCACGAAGTAG | RT-qPCR |
| **02157_qPCR_up** | TTCGGTGTCGACGAAGGATG | RT-qPCR |
| **02157_qPCR_down** | TCCGCCGTGCTCTTTTTGTA | RT-qPCR |
| **02156_qPCR_up** | TGCGATGGACCCACGACTAT | RT-qPCR |
| **02156_qPCR_down** | TCAGCGGTACCTCGGAGAGA | RT-qPCR |
