# Supplementary material for: Cross-regulation and cross-talk of conserved and accessory two-component regulatory systems orchestrate Pseudomonas copper resistance
Source: PLoS Genet. 2024 Jun 11;20(6):e1011325. doi: 10.1371/journal.pgen.1011325 (PMC11195947; doi:10.1371/journal.pgen.1011325)
Supplement: Attachment — Submitted filename: 240415_Ms CusRS_Reviewerss responses_Elsen.pdf [file pgen.1011325.s011.pdf]

***Reviewer #1:*** *If bacterial copper tolerance mechanisms have been extensively studied over the last decades, the integration of their underlying regulatory pathways remains unclear.*

*By using mutagenesis, transcriptional fusions and Cu sensitivity readouts, this study is addressing in clinical isolate of Pseudomonas paraeruginosa the potential crosstalks upon Cu stress between a regulatory system acquired via horizontal transfer and core regulatory elements present on the chromosome.*
*As a general comment, the lacZ reporter assays performed in this study suggest a cross-talk between two main regulatory networks. My main concern is the lack of supporting experiments and/or references (1) to the kinase/phosphatase activity of CopS and CusS and (2) to the competition between RR for promoter binding. In addition, some parts of the manuscript should be reworked in terms of structure and precision for a better understanding of the data and the message should be clarified.*

We thank the reviewer for his/her comments. Regarding the two main concerns :

1) We have added clearly the reference for the constitutive-phosphatase mutant $CopS_{H235A}$ of *P. aeruginosa* (Novoa-Aponte *et al*., 2020, mSphere) and performed new experiments by using mutants for kinase and phosphatase activities (see latter).

2) Genetic data support the binding of CopR and CusR on the same site. The fact that the outcomes can differ depending on the response regulators is not really new (for example, see CopR1/2 and CzcR in *P. stutzeri*. *Garber et al*., 2018, Mol Microbiol).

## Major comments

*Introduction*

*The introduction should provide more information on the regulatory pathways involved in copper resistance.*
*The authors should emphasize the biological question and the originality of their study*

We have added an information on CueR (page 5, lines 102-105). The functioning of CopRS is already described in detail within the Results section. We have modified the end of the introduction to introduce the biological question and to stress out the originality of the study.

*Results*

*Lines 130-133: the authors should clarify what they mean by "two divergently transcribed operons" and on which basis they consider these operons as "reminiscent of the copSR-copABGOFCDK locus identified in the soil-based heavy metal-tolerant β-proteobacterium Achromobacter sp. AO22".*

We now completed the sentence : «….:the gene organization is indeed similar and the sequence identity of the predicted proteins ranges from 84.9 to 99.7 % [26] (Fig 1A). ».

*Lines 136-139: the authors mention that "The regulatory genes IHMA87_02165-66 encode a putative copper-responsive TCS that shares homology with P. aeruginosa CopRS". Yet, they rename it as CusRS. Would it be possible that CusR is in fact another CopR? The authors did not mention the percentage of identity/similarity of CusR with CopR. Please clarify.*

We added the % identity with CopS and CopR and change the phasing to read:

"….we named this TCS CusRS due to the higher identity of the predicted proteins with the *E. coli* CusR and CusS (70.7% and 41.5% amino acid identity, respectively) than with IHMA87 CopR and CopS (69% and 36.2% amino acid identity, respectively".
Naming them CopR2 and CopS2 could have been misleading, making them paralogs, which they are not (HGT-acquired *cusRS*).

*The deltacopR mutant does not exhibit the same sensitivity to Cu in fig. 2A et fig.2B. How do the authors explain this difference? In addition, what could be the black colonies present in or at the periphery of the drops of fig. 2B?*

The differences in sensitivity between the mutant strains and the wild-type strain are consistent and have always been observed. However, we observed slight differences depending on the batch of medium, the size of the plastic plates (more or less large) and the time given to the colonies to grow. As far as the « black colonies » are concerned, it was a question of clarity and quality of the image.
To avoid any confusion, we have modified the **Figure 2B** and present the data obtained under exactly the same conditions as in Figure 2A, with 0 and 20 mM $CuSO_4$.

*Statistical analyses are missing in fig. 1D, fig. 1E, fig. 3A-E, fig. 5A-E, fig. S1, fig.S5 and fig. S8*

As requested, we added the statistical analyses on all the β-galactosidase data.

*Lines 322-333: Kinase/phosphatase mutants of CopS and CusS seem to be overstated unless they were previously experimentally tested. In this case, references should be added. Where do the mutants used in this study come from?*

We are sorry for ommiting previously the reference to the constitutive phosphatase activity of $CopS_{H235A}$ of *P. aeruginosa* in this paragraph (Novoa-Aponte *et al*., 2020, mSphere). It has been now added in the text. We ingeneered and experimentally tested the $CopS_{H235A}$ protein mutant. In *P. paraeruginosa* IHMA87 and obtained the expected phenotype (**Figure 5A**).

In the revised manuscript, based on the literature on HKs (Willett and Kirby, PLoS Genet, doi:10.1371/journal.pgen.1003084. Barr *et al*., Mol Microbiol., doi:10.1111/mmi.15019), we also generated phosphatase KO mutant, $CopS_{T239A}$. Our additional data presented in **new Figure 5F** are consistent with the model proposed by Novoa-Aponte *et al*., 2020.

In addition, we generated phosphatase-deficient protein mutant, $CusS_{T270A}$, of IHMA87 strain, and we observed phenotypes in consistent with a consensual TCS molecular functioning (**new Figures 5 G,H).**

*How to reconcile the EMSA data (Fig. 1C) performed with recombinant unphosphorylated His-CusR with the lacZ reporter assay claiming that the phosphorylation status of CusR is important for its function (lines 330-332)?*

The binding of CusR *in vitro* is not only specific, but also observed at a reasonable concentration of the protein (apparent equilibrium binding constant [Keq] of ~ 25 nM). The addition of acetyl phosphate did not increase the affinity of CusR for its target probe during EMSA, suggesting that the CusR does not use this molecule as a phosphodonor, which is common observation for RRs. Even *in vivo*, some RRs bind DNA and activate their targets as their concentration increases. This is often used by researchers to activate the RR when the signal that activates the TCS is unknown (some examples : doi: 10.1128/JB.183.4.1455-1458. 2001 ; doi: 10.1128/ JB.00387-06 ; doi : 10.2323/jgam.2020.01.004 ; doi : 10.1080/09168451 .2017.1350565).

Indeed, in our hands, increasing the concentration of both CusR and $CusR_{D51A}$ mutant proteins *in vivo* (in the *ΔcopSΔcusS* mutant, **Figures 5C,E**) leads to the binding of the transcription factor to its targets, as measured by activation of target gene expression.

*Lines 338-379: this section is quite confusing and should be reworked to facilitate the interpretation of the data and to highlight the main messages.*
The section has been reworked. But we had to keep the order of ideas to describe the panels A to E as the data was already there. However, we have generated new mutants and completed the description of the crosstalk by adding important results on HK phosphatase activities that strengthen our model.

*Discussion*

*474-475: the authors should discuss how Pco proteins maintain copper compartmentalization.*
We added the following information:
« Indeed, PcoA2 is a putative periplasmic multicopper oxidase oxidising Cu(I), PcoB is a putative outer membrane exporter protein, PcoD is an inner membrane protein and PcoF is a putative P-type ATPase that extrudes Cu from the cytosol. PcoC, PcoG and PcoK are predicted copper-binding periplasmic proteins that are likely to play a chaperone role, and PcoO may also be a chaperone but is located in the inner membrane. The Pco proteins can then be distributed in all compartments and membranes of the bacteria and participate in both periplasmic and cytosolic Cu detoxification, in particular by controlling Cu shuttling. »

*484-487: please discuss this point more extensively.*
We have added the following:
"We can hypothesize that the conserved copper resistance proteins controlled by the CopRS system are so efficient that an interaction between CopR and RNA polymerase has not been selected during evolution to activate the *pcoA2* operon since the acquisition of ICE. It is also possible that the fact that both CopRS and CusRS respond to the same signal (the same range of copper levels) did not prone CopR to activate expression of accessory genes already controlled by CusRS."

*491-493: the authors state that they confirmed that CopRS relies on a "phosphatase mechanism". They should discuss how copper can shut down CopS phosphatase activity.*
Unfortunately, we can not speculate more on the molecular mechanistics, as our work was based on *in vivo*, genetic analyses and not on biochemical data with purified proteins. This is probably why Novoa-Aponte *et al* (2020, mSphere), who proposed this phosphatase mechanism, did not do it either.

*524-528: please discuss the rationale of having 5 different Cu tolerance mechanisms.*
The lines already read: « Although generally considered to be a disadvantage for the bacteria, as it may decrease the specific response and trigger inappropriate responses, cross-talk can also be beneficial. Indeed, it can provide some evolutionary benefits, such as complex signal-processing mechanisms or priming to respond to future stimuli [44,49,50]. We added " Having four different TCSs for copper tolerance probably allows the IHMA87 strain to fine-tune the expression of genes encoding the appropriate copper-related systems. "

**Minor comments**

*Abstract*

*Line 32: "the entire locus" => which locus?*
Added : the *cusRS-pcoA2* locus.

*Introduction*

*The description of the PA7 strain used in this study should be more detailed :*

We added a sentence in the result part : « We have chosen the Exolysin-secreting clinical isolate IHMA87 as a model several years ago because, unlike PA7, this strain is virulent and amenable to genetic manipulation ».

*Lines 68-70: periplasmic chaperones do not provide Cu to P1B-type ATPases in the frame of Cu efflux.*

Thank you for pointing this mistake. The two sentences together were misleading. We modified the first one to read : « Periplasmic and cytoplasmic chaperones provide copper for cuproprotein biogenesis or deliver it to export systems. »

*Line 120: the authors should detail which accessory TCS they are referring to.*

Yes

*Results*

*Line 135: please specify which locus you are referring to.*

Done

*Line 231-233: I suggest the authors to add "alone" after "…these mutations".*

Added, as suggested

Lines 261-263: the authors should try to be less assertive.

We modified the sentence : "This observation <u>suggested</u> that CopR binding does not lead to transcriptional activation of the *pcoA2* promoter and, by competing for binding on the same palindrome, CopR <u>seems to limit</u> access for CusR, therefore reducing *pcoA2* activation".

*Line 266: please replace "expression" by "activation".*

OK

*Lines 298-299: please add "to Cu and Zn, respectively".*

Done

*Line 306: fig S5 instead of fig S6*

Done

*Line 312: fig S6 instead of fig S7*

Done

*Line 322: please add references for the constitutive phosphatase mutant (is it a H225A or H235A mutation? Both annotations are used).*

It is $CopS_{H235A}$ (corrected in **Figure 5**); we added the reference in the text.

*Line 332: please add references for CusR D51A (mentioned in the fig) or D57A (mentioned in the text).*

Asp54 was corrected into Asp51. We added the following information : « To determine whether this transcriptional activation was due to the activity of phosphorylated CusR, we mutated its phosphorylatable residue, Asp51, as in the CopR protein (Novoa-Aponte et al., 2020], and indeed observed that the signal was lost when non-phosphorylatable $CusR_{D51A}$ was synthesized in the Δ*cusS* background mutant."

*Lines 344-348: please rephrase in a clearer manner.*

We have rephrased as follows : « These data suggest that the HK CusS prevents *pcoA* gene expression surely by dephosphorylating CopR in the absence of both copper and CopS,

indicating that CusS can regulate CopR activity and compensate for an absence of the HK CopS. »

*The models should be moved to a main figure.*
The models have been moved now in two main figures to follow the text.

*Lines 371 and 375: please correct the cusR mutation.*
OK

*Materials and methods*
*Considering the importance of the lacZ reporter assay in this study, the authors should detail the protocol used.*
The protocol has been described in more details.

-----------------------------------------------------------------------------------------------------------------------------

***Reviewer #2:*** *Elsen and collaborators analyze the interplay between two copper signal-transduction regulators and a zinc response regulator on the expression of two horizontally acquired transcriptional units, cusRS, that encodes one of these regulators, and the divergent pcoA2B2GOFCDK operon. Interplays like this were previously reported, including the coordination between the copper and oxygen peroxide response regulators in E. coli (Urano et al, 2015), and a work in Pseudomonas stutzeri RCH2 by Garber et al (2018) that showed the interaction of three two-component regulators, CopR1, CopR2 and CzcR, and their control of copper and zinc homeostasis.*

We thank the reviewer for all his/her comments. The novelty of our work, which was maybe not emphasized enough, was not that there is a cross-regulation between CopR and CzcR, two conserved regulators of *P. aeruginosa* and *P. paraeruginosa*, but the discovery that an acquired TCS is fully integrated into the regulatory network and the cross-talk between the proteins of the acquired CusRS and conserved CopRS, two TCSs with different molecular functionning.
We would like to emphasise that our conclusions are not speculative but are inferred from data from our genetic approach. However, even though no *in vitro* molecular studies were performed in the paper, our models are based on years of literature on TCS studies.
Concerning the crticism that « This manuscript requires a major language use and grammar edition », we would like to point out that original manuscript was fully edited by a professional translator from TWS Editing Scientific writing.

***Major points***
**1.-** *In this manuscript, the authors showed that CusR controls the Cu-induced transcription of the pcoA2 operon and partially its own transcription. This locus is dispensable for resistance to the metal ion in the wild-type strain, but it provides some resistance in the absence of CopR, and/or the CopR-regulon proteins (shown in Fig 2 and in Fig S3A). With the information provided, and agreeing with the authors' indication (lines 234-236) that the absence of this locus has no relevance in Cu-resistance under the tested conditions, their conclusion that the proteins encoded in this locus are involved in copper detoxification and resistance is overstated at this point.*
We disagree with the reviewer. We have shown that the proteins encoded by the *pco* operon are able to confer Cu resistance in the absence of CopR. So, their effects are real, but masked at least in the laboratory conditions by the Cop system. Up to now, we did not find the conditions under which they could provide a growth advantage. We were very clear on this.

*Different physiological conditions under which the pcoA2 operon would provide some growth advantage will be tested to claim that this locus provides an advantage in the presence of excess copper.*

We agree and we did try different conditions (various media, urine…). However, we only found that they all confer an advantage in a CopR-deficient strain. We would like to point out that the aim of our work was not to identify the conditions under which the proteins encoded by the *pco* operon confer a growth advantage in the presence of a Cu stress, a search that could be very tedious.

***2.-*** *The conclusion that CopR, CusR and CzcR recognize the same target sequence needs to be demonstrated by alternative methods than DAP-seq, that only delimit a DNA region.*

DAPseq, like ChIPseq, is a high-throughput method for the discovery of TF binding sites. Both methods are based on the sequencing of DNA fragments bound to proteins. In other studies, such as our work on the ErfA inhibitor (Trouillon et al., 2020, NAR, doi: 10.1093/nar/gkz1232), we have observed that the summits of the peaks obtained by DAPseq corresponds closely to the binding sites. Furthermore, we also provided evidence *in vivo* by introducing mutations in the palindrome of the binding site and showing the loss of regulation by the three RRs (**Figure 1** and **new Figure 4B**).

*It can also by that of overlapping binding sites in the curR-pcoA2 intergenic region are recognized by these proteins until the identification of each regulator target sites.*

The mutation of the binding site resulted in the loss of Cu regulation by CusR and CopR, and of Zn regulation by CzcR as now added to the new **Figure 4B** of the revised manuscript.

*The Escherichia coli CusR protected region encompasses a AAAATGACAAn(2)TTGTCATTTT dyad sequence.*

Yes, we agree. We indeed wrote in the manuscript « *E. coli* CusR preferentially binds to the palindrome "AAAATGACAAnnTTGTCATTTT" [Munson *et al*., 2000], located between the two divergent operons *cusRS* and *cusC(F)BA*."

*In Pseudomonas, at least three predicted CopR binging sites were reported. Quintana et al (2017) showed a TGACAn(4)TGTaAT conserved motif, and the authors of this manuscript previously reported a GTCAGn(6)GTCAG direct repeat (Trouillon et al, 2021). None of these coincides this manuscript´s postulated binding site, CTGACAn5GTAAT. It is clear that the identification of the sequence recognized by each of the three regulators analyzed in this manuscript is required.*

Unfortunately, we must say that we disagree with this statement.

Firstly, our « postulated binding site » of CopR is the one found in the PRODORIC database « CTGACAn5GTAAT », based on the data of Quintana *et al*., 2017 (TGACAn4TGTaAT) - very consistent.

Secondly, in Trouillon *et al.,* 2021, the binding site was determined on the basis of all the peaks obtained in DAPseq, even those resulting from an excess of proteins - it is therefore less accurate, as often the case with high-throughput methods.

Finally, as mentioned above, the mutation of the half-site "GTAAT" in "AGCCT", half-site conserved in the binding consensus of the three RRs (see below), led to the loss of Cu regulation (**Figure 1**) and Zn regulation (**new Figure 4B**).

[CusR: ATTCATnnATGTAAT    CopR: CTGACA$n_5$GTAAT    CzcR: GAAAC$n_6$GTAAT]

**3.-** *Their claim that the same cusRS promoter binding site is required for the activation by both CusR and CopR in vivo is at the least overstated until proven. Although the authors showed that in a cusR mutant CopR can induce cusRS transcription in the presence of Cu, this*

*"ectopic" activation can be caused because of the absence of the innate regulator that will leave a target site free to be occupied by a lower affinity regulator.*
We agree with the Reviewer that it is all a matter of dynamics and depends on protein concentration, protein phosphorylation and DNA affinity. We do not exaggerate what can be inferred from the data obtained with our genetic approach. The experiments clearly indicated that both CusR and CopR can bind to the same sequence *in vivo* and that the Cu response is lost when the binding site is mutated.

*To confirm that both transcription factors act in vivo on the control of cusRS transcription, experiments like ChIP are required. This will show that both regulators occupy the cusRS promoter region inside the bacterial cell in the wild-type strain.*
We do not believe that this would add value and change the message of our study. Our data are supported by an *in vivo* approach, based on transcriptional fusions coupled with the use of numerous mutants, and are supported by our previous DAPseq.

*In this regard, which would be such a physiological situation in which active CopR, induced by the metal ion, interacts with the cusRS promoter but there is no enough active CusR to interact with its target site?*
We absolutly agree that it would be interesting to determine the physiological signal, but this goal is out of the scope of our work, which is more focused on the CopRS-CusRS cross-talk.

***4.-** The authors sowed that CusR exerted a positive regulation on both target transcriptional units while CopR functions as an activator of cusRS and at the same time as a repressor of the pcoA2 operon. Considering that both regulators recognize the same binding site, what is the explanation for the antagonistic behavior of CopR on these targets?*
The explanation could be the binding but no interaction with RNA polymerase, as can be observed with some transcription factors. To illustrate it, we already mentioned in the text « …these variable outcomes may also be due to differences in RR-DNA and/or RR-RNA polymerase interaction, as demonstrated for the *E. coli* paralogs KdpE and OmpR (Ohashi *et al*., 2005)".

***5.-** The observation that in the presence of Zn CzcR can modulate the expression of both cusR and pcoA2 promoters is interesting, although it resembles what was reported in P. stutzeri RCH2 (Garber et al, 2018).*
Indeed, as we mentioned it several times in the manuscript.

In this regard, is CusR required for this modulation?
It seems that the answer is no, based on our data (loss of Zn-response in Δ*czcR* mutant).

*Is this response affecting zinc homeostasis/resistance? Are the proteins encoded in the pcoA2 operon involved in Zn homeostasis? And finally, what is the Zn-resistance phenotype of the mutant deleted of this operon?*
We did not observe any influence of the proteins encoded by the *pcoA2* operon on the Zn resistance in our experimental assays (Zn sensitivity plate assays in M9 and LB), consistent with their predicted role in copper resistance. This is different from what was observed for the *ptrA-czcE-queF* operon, as mentioned in the discussion. We have decided not to show these results as they are, in our opinion, no relevant. *In sum, is this regulation relevant for this bacterium physiology?*

In the study on *P. stutzeri* (Garber *et al*., 2018), the authors suggested that the « cross-regulation of copper and zinc homeostasis can be advantageous ». We agree, and we mentioned in the discussion that such regulation could be relevant, as the two ions could be found under the same physiological conditions. However, the aim of our paper was not to

identify the conditions under which this regulation is <u>relevant to bacterial physiology</u>, a search that could be very tedious.

***6.-** Sentences from lines 366 to 369. What do the authors mean with the following observation? "Copper-blind expression of both PcusR and PcopA2 was then observed in the ΔcusSΔcopS mutant. As CusR cannot be phosphorylated in bacteria lacking both sensors (CusS and CopS), the PcusR expression pattern observed (Fig 5D) can be attributed mainly to dysregulated CopR activity (Fig 5C)." How is CopR active then?*

Novoa-Aponte *et al*., 2020 proposed, based on their experimental data that CopR is activated by another element that has not yet been identified. To make this clear, we have added in the text together with the reference : "The mechanism leading to the phosphorylation of CopR remains unclear, as the phosphodonor has not yet been identified" (page 15, lines 339-40).

The authors did not determine the concentration of any of the regulators analyzed in this manuscript.

Indeed, as our work relies on genetic experiments.

Then, to indicate that "high levels of unphosphorylated CusR also contributed to these activities as the copper-blind expression of P*cusR* noticed in CusRD514AΔcopSΔcusS was still observed, albeit to a lesser extent (Fig 5D)" and the following asseveration is pure speculation.

We disagree with the reviewer's comment, as these conclusions are drawn from our results : **Figures 5D** and **5E** show similar activities of the transcriptional *lacZ* fusions in the Δ*copS*Δ*cusS* producing either wild-type CusR, which can be phosphorylated, or CusRD51A mutant protein, which cannot be phosphorylated : this suggets that the transcriptional activity is not due to phosphorylated CusR but to overproduced CusR (due to constitutive CopR activity on *cusRS* promoter in the double mutant), which can bind and activate transcription as, mentioned in the text. However, we have modified our description of these two figures to be clearer.

***<u>Minor points:</u>***

*This manuscript requires a major language use and grammar edition.*

We are quite surprised by this comment as the original manuscript was fully edited by a professional.

*Lines 176-177. As the actual binding site of CusR was not identified yet, the conclusion made in this sentence is overstated.*

We disagree as the binding site was identified by DAPseq, it is clearly similar to those of CopR and CzcR, and its mutation leads to the loss of Cu and Zn regulation.

*Sentence beginning in line 363. As the experiments showed in this manuscript were done using lacZ transcriptional fusions that CusS controls the phosphorylation status of CusR and of CopR it is just speculative. The phosphorylation status of these proteins was not determined in this work.*

We agree with the last sentence because all our experiments are based on a genetic approach and not on *in vitro* molecular studies. However, our conclusions are not speculative but based on our knowledge of years of literature on TCS studies (amino acids involved in kinase and phosphatase activities, phosphorylation sites…).

Fig. 1B. What does the arrowhead over the last T that the authors marked as a CusR binding site mean?

The arrowhead corresponds to the summit of the peak identified by previous DAPseq, as indicated in the figure legend.

*Fig. 1D and E. The authors should indicate the precise sequence of each promoter construct.*

The lengths of the fragments are given in S1 Table. To give them in the figure would make it heavier and would not add any further information, as the fragments are drawn to scale and the important features are also shown.

*Fig. 2. It would be more informative to test other Cu concentrations to determine the MIC for each strain.*
We do not believe this is relevant to our study.

*Fig. 3. Could the authors have any explanation about the differences in Cu-sensitivity observed in these experiments and those shown in Fig. 2?*
- **Figure 3** : All the β-galactosidase assays in each panel were performed on the same day and on cultures treated in the same way (growth, LB, $OD_{600}$), as these conditions can slightly affect the activity levels, but **never** the differences observed between the strains, which were always maintained.
- **Figure 2** : This is exact. The differences between the strains are consistent and have always been observed ; however, depending on the batch of medium, the size of the plastic plates (larger or smaller) and the time given to the colonies to grow, we were able to observe slight differences between the experiments. To avoid any confusion, we have decided to modify **Figure 2B** and present the data obtained under exactly the same conditions as in **Figure 2A.**

*Statistical significances should be provided for this as well as all the transcriptional analyzes made in this work.*
We have added the statistical significance for all β-galactosidase data where relevant.

*Fig 5. Please, correct the figure legends as some mutant strains are wrongly named.*
Thanks for this point.

---

***Reviewer #3:*** *The manuscript by Elsen et al. characterizes the role of the lysR-cusR locus in copper resistance by Pseudomonas aeruginosa strain IHMA87, and its regulation by several two-component systems, most notably by the CusRS, CopRS, and CzcRS systems. The authors showed that CusR and CopR are important for copper stress resistance. Through extensive and elegant genetic analyses, they also demonstrated that the CusRS and CopRS both regulate the expression of cusR and pcoA2 locus. Overall, the experiments were well designed and the main conclusions above are supported. The study contributes to our understanding of copper resistance among P. aeruginosa clinical strains. However, because the study undertook only the genetic approach and analyzed only kinase-deficient mutants of CusS and CopS, the molecular mechanisms of cross-talk need some refinement. Bringing some schematics to the main figures will also help the readers through complex molecular interactions.*

We would like to thank the reviewer for her/his appreciation of our work and for her/his pertinent and valuable comments, which allowed us to strengthen the *in vivo* cross-talk model we initially proposed based on the results of our genetic experiments.
As requested, we now provide the data that strongly support the *in vivo* interaction between CusS and CopR (analyses of the Δ*copS*Δ*cusR* mutant), which nicely complements those already shown on CopS-CusR (analyses of the Δ*cusS*Δ*copR* mutant). In addition, we have constructed and analysed the phosphatase-negative CopS and CusS HKs. The phenotypes are consistent not only with the phosphatase-based mechanism of the CopRS system proposed by Novoa-Aponte *et al*. (2020), but also with the control of phosphorylation state of CopR and CusR, and consequently their effect on the expression of their targets.

***Major scientific comments:***

**1.** *The role of CusR regulon in copper resistance (Fig. 2):*

- *I find it confusing why the ΔcopR pcoA2::Ω strain is more sensitive to copper stress than ΔcopR, given that CopR is a repressor of the pcoA2 locus, and that the pcoA2::Ω strain itself is not sensitive. Please include an explanation in the Discussion section.*

We have already explained in the text that the proteins encoded by the *pcoA2* operon are capable of conferring resistance to the bacteria, but that their effect is masked by those of the CopR regulon, which efficiently detoxify the bacteria. If the main activator of the operon, CusR, is inactivated, there is no effect either. The regulatory effect of CopR on the locus is minimal, as it slightly reduces the *pcoA2* expression in LB media.

- *Because pcoA2::Ω further increases copper sensitivity of ΔcusR ΔcopR, this would suggest that pcoA2 is expressed in the ΔcusR ΔcopR strain. But this would contradict gene expression results shown in Fig. 3B. Please explain this incongruence.*

The expression of *pcoA2* in the *ΔcusRΔcopR* strain is indeed minimal in the strains grown in liquid LB (**Figure 3B**). However, for the copper sensitivity plate assay, bacteria are grown on plates and minimal M9 medium, so the basal level of *pcoA2* expression is likely to be sufficient to confer some level of copper resistance.

However, in the revised manuscript, we have changed **Figure 2B** (with both **Figures 2A** and **2B** showing the data with 0 and 20 mM $CuSO_4$) following a comment from another reviewer. It was observed that there was a difference in the copper resistance of the Δ*copR* mutant between panels A and B and also that black colonies seemed to be present in or at the periphery of the drops of **Figure 2B**. All these observations are valid, but it should be emphasized that the differences in copper resistance between strains are always consistent and have always been observed. On the other hand, depending on the batch of medium, the size of the plastic plates (larger or smaller) and the time given to the colonies to grow, we observed some slight differences from one experiment to another. To avoid any confusion, we decided to modify **Figure 2B** and present the data obtained under the same conditions as in **Figure 2A**. Regarding the black colonies, this was simply a question of clarity and quality of the image, which has been corrected.

- *Given the ambiguity of the phenotypes related to pcoA2::Ω disruption, complementation of this locus should be introduced into disrupted strains and examined for copper sensitivity.*

We do not think that the phenotypes of the *pcoA2*::Ω mutants are ambiguous, but we agree with the Reviewer on the value of complementation. However, although the strain IHMA87 is more amenable to genetic manipulation than the reference strain PA7, we still cannot efficiently introduce and maintain replicable plasmids in it. Only integrative plasmids (such as mini-CTX) or suicide plasmids can be used. For example, although mini-CTX plasmids can be transferred into bacteria and integrated into the *att* site of the chromosome, its backbone cannot be excised with the pFLP2 replicative plasmid (Hoang *et al*., 1998 ; DOI: 10.1016/s0378-1119(98)00130-9), which explains why all the *lacZ* strains are resistant to tetracycline.

**2.** *Transcriptional regulation of cusR-pcoA2 by CusRS and CopRS (Fig. 3)*

- *In the presence of CusR (WT background), the effect of copR deletion is rather small for both cusR and pcoA2 expression. Please perform statistical analyses and comment on the physiological relevance of CopR in cusR-pcoA2 expression.*

The statistical analyses have been added, but the consistency of the observations and measurements is, in our opinion, more important.

The effect of *copR* deletion is indeed « small » but real and consistent with the binding previously observed *in vitro* by DAPseq. We do not know if there is a physiological relevance

(fine-tuning of the Cu response ?) or if this is just due to the similarity of the binding sites of the Cu-responsive RR.
We added a comment on the CopR action in the discussion: « We can hypothesize that the conserved copper resistance proteins controlled by the CopRS system are so efficient that an interaction between CopR and RNA polymerase has not been selected during evolution to activate the *pcoA2* operon since the acquisition of ICE. It is also possible that the fact that both CopRS and CusRS respond to the same signal (the same range of copper levels) did not prone CopR to activate expression of accessory genes already controlled by *cusRS*.”.
- *Why is the ΔcusR ΔcopR strain completely abolished for copA1 expression (Fig 3C)?*
The mistake has been corrected, it is actually the Δ*cusR*Δ***cueR*** strain, not Δ*cusR*Δ***copR***.

**3.** *Regulation of cusR-pcoA2 by other response regulators*
- *The authors' conclusion about binding of other response regulators to cusR-pcoA2 region is solely based on DAPseq data, which would need to be validated by independent methods. I'm not asking the authors to perform validation experiments in this study, but please consider the possibility of false-positive results from DAPseq.*
False-positive results from DAPseq are indeed possible, as is often observed with high-throughput approaches and as we have experienced many times. However, we strengthen our « cross-regulation model » by showing in the **new Figure 4B** that mutation of the identified binding site also leads to loss of regulation by CzcR (loss of Zn induction of *pcoA2* and *cusR* expression). This is in addition to the complete loss of Cu regulation of the *cusR* promoter, which we have shown to be normally activated *in vivo* by both CusR and CopR. Therefore, all the *in vivo* data strongly support the DAPseq data.

- *Is there an explanation for why the ΔczcR strain exhibits increased cusR expression in response to Zn + Cu (Fig. 4, right panel)?*
No, not really. We also observed a small increase in this mutant for P*czcC-lacZ* but not for P*pcoA2-lacZ*. So we prefer not to speculate.

**4.** *Cross-talk between the CopRS and CusRS systems (Fig. 5): The genetic analyses are elegant but at some points are unnecessarily complex and therefore can be confusing to the readers.*
- *The constitutive expression of pcoA and cusR in the ΔcopS ΔcusRS strain indicates that CopR is phosphorylated by another factor, so it is surprising that the kinase-deficient CopS mutant results in complete abolishment of pcoA expression (Fig. 5A).*
In fact, as written in this part, it has been published that the $\text{CopS}_{\text{H235A}}$ is a constitutive phosphatase (Novoa-Aponte *et al.,* 2020), explaining the absence of CopR activity.

*Therefore, a phosphatase-deficient CopS mutant will be extremely useful to directly demonstrate the phosphatase-dominant function of CopS.*
Indeed, and we generated a phosphatase-deficient CopS ($\text{CopS}_{\text{T239A}}$). The new data are now shown in **Figure 5** (**Figures 5 F-H**) and are consistent with our model.

- *Does CusS interact with CopRS: I agree with the authors' interpretation of Fig 5C that CusS regulates CopR. However, this data does not indicate that CusS can dephosphorylate CopR.*
In fact it does, and we have demonstrated it (see below).

*Specifically, constitutive pcoA expression in the ΔcopS ΔcusS strain indicates that something else can phosphorylate CopR, and the loss of expression in the copS H235A ΔcusS strain suggests that CusS can phosphorylate CopR, as an alternative interpretation to the authors' conclusion.*
However, $\text{CopS}_{\text{H235A}}$ is a constitutive phosphatase (Novoa-Aponte *et al*., 2020).

*A phosphatase function of CusS can be directly demonstrated with a phosphatase-deficient mutant.*
As suggested, we have generated and analysed mutants that produce a phosphatase-deficient CusS protein ($CusS_{T270A}$). The data strengthen our model - CusS can act as a phosphatase towards CopR in the absence of CopS and in the absence of Cu (**new Figures 5 F,G,H**).

***5.*** *Does CopS interact with CusRS (Fig 5D-E): Given that cusR and pcoA2 expression is also regulated CopR, the authors' interpretation of whether CopS regulates CusR can be confounded. Therefore, interaction between CopS and CusR would be most directly demonstrated in a ∆cusS ∆copR strain.*
We agree that these data were clearly missing. They are now included in the **new Figure 5** (**Figures 5 G,H**), and they strengthen the model.

***6.*** *Statistical analyses are lacking in most figures.*
We have added the statistical significance for all β-galactosidase data.

***<u>Minor comments:</u>***

\- *Given the complex cross-regulation and cross-talk mechanisms, it will be helpful to move Figure S6 and a simplified version of Fig S7 to the main figures.*

We have moved **Figure S6** and **Figure S7** in the main text, but we were unable to simplify **Figure S7**, which already doesn't include the different mutants for kinase and phosphatase activities.
