# Supplementary material for: Cross-regulation and cross-talk of conserved and accessory two-component regulatory systems orchestrate Pseudomonas copper resistance
Source: PLoS Genet. 2024 Jun 11;20(6):e1011325. doi: 10.1371/journal.pgen.1011325 (PMC11195947; doi:10.1371/journal.pgen.1011325)
Supplement: Attachment — Submitted filename: 240523_Ms CusRS_Elsen_Reviewers responses.docx [file pgen.1011325.s012.docx]

PLOS Genetics (PGENETICS-D-24-00418)

Reviewer #1: In the previous version of this manuscript, the authors reported extensive cross-talks between the two component systems CusRS and CopRS. In this revision, the authors used phosphatase-deficient mutants of CusS and CopS to demonstrate their phosphatase activities towards cognate and non-cognate response regulators. These analyses much strengthen the model and the authors have addressed all my previous questions. However, there remain two minor points:

1. Please put the pcoA2::Ω strain back to Figure 2B. This is an important control for the ΔcusR and ΔcusR pcoA2::Ω phenotypes, because pcoA2 is regulated by multiple transcription factors.

We agree with the reviewer’s comment and we have reinstated the figure provided in the first submission, showing the results at 0-5-20 mM CuSO_4_ for all the strains, including the pcoA2::Ω and ΔcusRΔcopR pcoA2::Ω strains. Therefore, we have added the sentence : Lines 224-225 « This sensitivity was observable at 5 mM CuS0_4_ and accentuated at the highest concentration (Fig 2A), although the magnitude of the effect may vary somewhat between replicates, as shown in Fig. 2B ». In addition, we have added a sentence regarding the increase in Cu sensitivity upon introduction of pcoA2::Ω into the double mutant, which was highlighted by a previous comment : Lines 232-235 « We can also see that *pcoA2*::Ω further increases the copper sensitivity of ΔcusRΔcopR, suggesting that the basal level of *pcoA2* expression in minimal medium and on plate is probably sufficient to confer some degree of copper resistance. »

2. Lines 440-445 still do not sufficiently explain why, in the ΔcusS ΔcopR strain, PcusR-lacZ and PpcoA2-lacZ is reduced by half in response to copper. It does appear that CopS can phosphorylate CusR, as written in the text. But why would basal expression be higher than copper-induced expression?

We agree with the reviewer that this is puzzling. So far, only the phosphatase activity of *P. aeruginosa* CopS towards CopR has been documented (Novoa-Aponte et al., 2020), an activity that is switched off upon copper detection : our data (Fig 5A,F) are also consistent with this model. However, our data provided additional information and clearly showed that CopS also has an autokinase activity, since CusR can be activated by CopS through phosphotransfer (Fig 5G,H). Unexpectedly, this kinase activity appears to be partially reduced upon copper detection. The unorthodox behaviour of CopS may be related to the fact that its kinase activity is not required for CopR activity and normal expression of the CopR regulon (upregulation in the presence of copper). Of course, it will be very interesting to investigate the molecular properties of CopS activities *in vitro* but this is beyond the scope of our paper. However, we have added a sentence in the text to mention this point.

Lines 450-453 “Interestingly, copper seems to partially decrease rather than increase this activity. Although non-intuitive, unorthodox regulation of the kinase activity may be related to the fact that it does not seem to be relevant for the function of the TCS CopRS and the expression of the CopR regulon.”

Reviewer #2: The authors addressed the concerns raised in my review of the former version of their manuscript (PGENETICS-D-23-01065). The message of the paper is now clearly stated and the explanations are more clear.

We thank the reviewer for her/his appreciation of our work.
